# Oral Pharmacokinetic Evaluation of a Microemulsion-Based Delivery System for Novel A190 Prodrugs

**DOI:** 10.3390/biom15081101

**Published:** 2025-07-30

**Authors:** Sagun Poudel, Chaolong Qin, Rudra Pangeni, Ziwei Hu, Grant Berkbigler, Madeline Gunawardena, Adam S. Duerfeldt, Qingguo Xu

**Affiliations:** 1 Department of Pharmaceutics, Virginia Commonwealth University, Richmond, VA 23298, USA; poudels4@vcu.edu (S.P.); cq87@yahoo.com (C.Q.); pangenir@vcu.edu (R.P.); gunawardenam@vcu.edu (M.G.); 2 Department of Medicinal Chemistry, University of Minnesota, Minneapolis, MN 55414, USA; huziwei2008@gmail.com (Z.H.); berkb004@umn.edu (G.B.); 3 Department of Ophthalmology, Department of Pediatrics, Department of Biomedical Engineering, Center for Drug Discovery, Center for Pharmaceutical Engineering, and Massey Cancer Center, Virginia Commonwealth University, Richmond, VA 23298, USA

**Keywords:** PPARα agonist, bioconversion, oral bioavailability, oral delivery, permeability

## Abstract

Peroxisome proliferator-activated receptor alpha (PPARα) is a key regulator of lipid metabolism, making its agonists valuable therapeutic targets for various diseases, including chronic peripheral neuropathy. Existing PPARα agonists face limitations such as poor selectivity, sub-optimal bioavailability, and safety concerns. We previously demonstrated that A190, a novel, potent, and selective PPARα agonist, effectively alleviates chemotherapy-induced peripheral neuropathy and CFA-induced inflammatory pain as a non-opioid therapeutic agent. However, A190 alone has solubility and permeability issues that limits its oral delivery. To overcome this challenge, in this study, four new-generation ester prodrugs of A190; A190-PD-9 (methyl ester), A190-PD-14 (ethyl ester), A190-PD-154 (isopropyl ester), and A190-PD-60 (cyclic carbonate) were synthesized and evaluated for their enzymatic bioconversion and chemical stability. The lead candidate, A190-PD-60, was further formulated as a microemulsion (A190-PD-60-ME) and optimized via Box–Behnken design. A190-PD-60-ME featured nano-sized droplets (~120 nm), low polydispersity (PDI < 0.3), and high drug loading (>90%) with significant improvement in artificial membrane permeability. Crucially, pharmacokinetic evaluation in rats demonstrated that A190-PD-60-ME reached a 16.6-fold higher Cmax (439 ng/mL) and a 5.9-fold increase in relative oral bioavailability compared with an A190-PD-60 dispersion. These findings support the combined prodrug-microemulsion approach as a promising strategy to overcome oral bioavailability challenges and advance PPARα-targeted therapies.

## 1. Introduction

Peroxisome proliferator-activated receptors (PPARs) are a group of ligand-activated transcription factors that belong to the nuclear hormone receptor superfamily [1]. PPARs function as a heterodimer in combination with a co-activator complex, which binds to specific DNA sequences known as peroxisome proliferator response elements (PPREs) located in the promoters of target genes. This interaction regulates gene expression through transactivation or transrepression, leading to the activation or suppression of specific genes [2,3]. In the absence of ligands, these heterodimers bind with a co-repressor complex that blocks gene transcription. Pharmacologically, there are three subtypes of PPARs: PPARα, PPARγ, and PPARβ/δ. Although these isoforms share high similarity in ligand binding domain, their tissue distribution, biological function and targeting differ [4].

PPARα regulates lipid metabolism, inflammation, and glucose homeostasis and has emerged as a therapeutic target for hyperlipidemia, diabetic microvascular complications, liver diseases (MASH and MAFLD), and pain [5,6,7,8]. Fenofibrate, an FDA approved and commonly prescribed PPARα agonist, demonstrates efficacy in many of these indications, but suffers from limitations such as rapid hydrolysis to its active metabolite, fenofibric acid, which exhibits low receptor affinity, poor selectivity across PPAR subtypes (α, γ, δ), and dose-dependent toxicities (e.g., renal injury, pancreatitis) [9]. While newer agents like Pemafibrate (approved in Japan for the treatment of hyperlipidemia) exhibit favorable safety profiles and hold therapeutic promise, their lack of approval in the U.S. highlights the need for the development of novel selective PPAR-α agonists [10,11]. Furthermore, preclinical limitations of these existing therapies, including suboptimal bioavailability and hepatotoxicity risks in rodents, underscore the demand for molecules with enhanced pharmacological properties [12,13].

**Figure 1:**
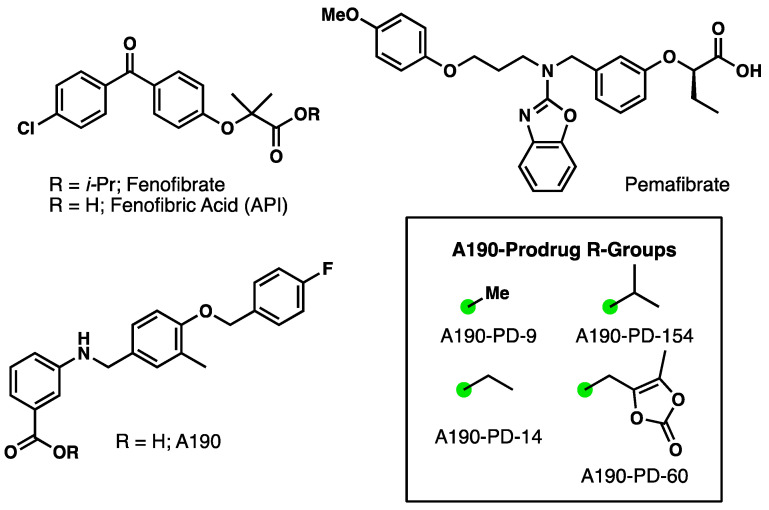
Structures of molecules discussed. The green dot represents the attachment point of the R-Groups to A190.

With newfound interests in novel PPARα agonists, especially non-fibrates, our team has developed A190. This molecule belongs to the 4-benzyloxy-benzylamino chemotype in which a novel benzoic acid pharmacophore has been substituted for the typical fibrate headgroup (Figure 1). A190 exhibits high potency (EC_50_ < 40 nM) and exceptional subtype selectivity (>2700-fold for PPAR-α over γ/δ isoforms) [14,15]. Preclinical assessment of A190 reveals acceptable microsomal metabolic stability and absence of CYP450 and hERG-related cardiotoxicity, positioning it as promising active pharmaceutical ingredient (API) [9]. However, therapeutic potential of A190 is hindered by poor aqueous solubility (0.028 mg/mL) and low intestinal permeability which limit its oral bioavailability [16].

Despite being the preferred administration route, oral drug delivery systems often encounter issues such as inadequate solubility and permeability, gastrointestinal breakdown, and first-pass metabolism [17,18]. Consequently, newer strategies emphasize leveraging novel nanotechnology platforms to improve upon these limitations [19,20]. Microemulsions, for instance, are increasingly employed due to their allowance for high drug content, micro-sized droplets, and spontaneous dispersibility–potentially improving drug uptake [19,21]. Microemulsions are thermodynamically stable systems formulated with an optimal composition of oils, surfactants and co-surfactants. Among the vast advantages of microemulsions are self-emulsification, simplicity in preparation, and scalability. Formulated with optimized oil derivatives and stabilized by surfactant/co-surfactant blends, microemulsions are able to enhance the oral bioavailability of hydrophobic drugs by prolonging gastrointestinal transit, boosting lymphatic transport, improving intestinal permeability, and inhibiting metabolic/efflux processes [22,23,24].

To overcome the limitations with A190, our team recently reported a microemulsion-based delivery system comprised of 7.5% of oleic acid and a Tween 80/PEG 400 ratio of 1:1 *v*/*v* which significantly enhanced the bioavailability (~5-fold) of A190 following oral administration [16]. The resulting formulation (A190-ME) effectively reduced mechanical hypersensitivity in a chemotherapy induced peripheral neuropathy (CIPN) mouse model in a PPARα dependent fashion and was also found to be equally effective at reducing chronic inflammatory Complete Freund’s Adjuvant (CFA)-induced pain. This study highlighted the potential of A190 to overcome the limitations of traditional fibrates while offering a scaffold for PPARα-targeted therapies in neuropathic pain disorders.

**Figure 2:**
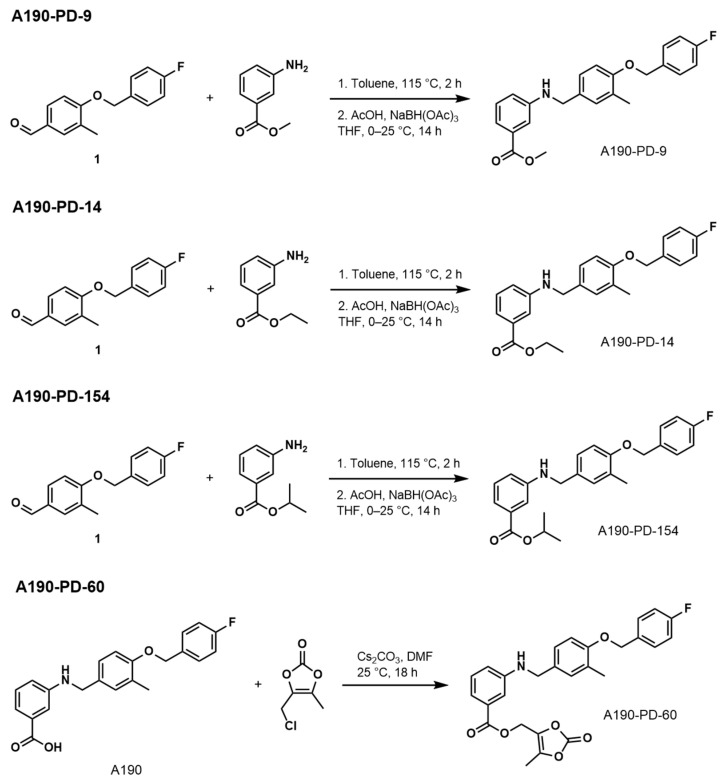
Synthesis of A190 prodrugs used in this study.

In this study, we synthesized and evaluated microemulsion formulation from four different A190 ester prodrugs (Figure 2). The rationale for investigating A190 prodrugs as microemulsion formulations arose from the following precedent: (1) Fenofibrate is administered as a prodrug (*i*-propyl ester) and its metabolism to the active API fenofibric acid illustrates the potential of PPARα agonist prodrugs, (2) A190 contains a free benzoic acid, a motif known to present metabolic liabilities, especially in the context of phase-II conjugation (e.g., glucuronidation), and (3) Microemulsion efficiency is expected to improve with higher hydrophobicity, a physicochemical change that will occur upon masking carboxylic acid. (4) In order to determine the best-performing candidate regarding conversion to parent drug and overall chemical stability. Due to the inherently higher hydrophobicity of the ester prodrugs compared to the parent A190 compound, we hypothesized that incorporating A190 prodrugs into a novel microemulsion formulation would enable the assessment of these more hydrophobic prodrugs for oral delivery. To test this hypothesis, we leveraged microemulsion technology to encapsulate the lead prodrug candidate (A190-PD-60) [(5-Methyl-2-oxo-1,3-dioxol-4-yl)methyl3-((4-((4-Fluorobenzyl)oxy)-3-methylbenzyl)amino)benzoate]. Subsequently, we conducted studies to evaluate the oral pharmacokinetics of the lead prodrug microemulsion (A190-PD-60-ME) to determine if this approach improved drug loading capacity and the oral pharmacokinetic profile of A190.

## 2. Materials and Reagents

For synthesis, reagents and ACS grade solvents were purchased from commercial sources and used without further purification. Reverse osmosis water was used for all experimental procedures where “water” is indicated. Analytical thin-layer chromatography (TLC) was performed on silica gel 60 F_254_ plates (Sigma-Aldrich, 1.05715, St. Louis, MO, USA). Flash column chromatography was carried out on silica gel (70-230 mesh, SiliCycle). ^1^H NMR spectra were collected on a 400 MHz NMR instrument. ^13^C NMR spectra were collected on a 100 MHz NMR spectrometer with complete proton decoupling. NMR data were collected at 25 °C. All NMR data were processed in MestReNova (Mestrelab Research, Santiago de Compostela, Spain). High-resolution mass spectrometry was obtained from and analyzed by the Mass Spectrometry Facility at the University of Minnesota using electrospray ionization. Diethylene glycol monoethyl ether (Transcutol HP), and propylene glycol monocaprylate (Capryol 90) were generously provided by Gattefossé (Saint-Priest, France). Tween 80 and Polyethylene glycol 400 (PEG 400) were purchased from Sigma Aldrich. Parallel artificial membrane permeability assay (PAMPA) plates were purchased from Thermofisher Scientific (Waltham, MA, USA). All LCMS and HPLC solvents were purchased from VWR (Radnor, PA, USA). Caco-2 cells (human colon cancer) and HepG2 cells (human hepatocellular carcinoma) were obtained from American Type Culture Collection (ATCC^®^, Manassas, VA, USA).

### 2.1. Prodrug Synthesis and Characterization

The detailed steps for synthesis of the respective prodrugs are outlined below. ^1^H-NMR and ^13^C-NMR characterizations for each are presented in Supplementary Materials.

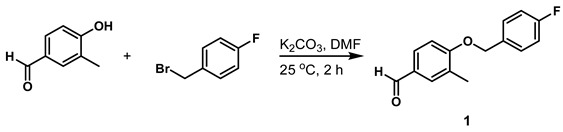

4-((4-Fluorobenzyl)oxy)-3-Methylbenzaldehyde (Compound 1)

A mixture of 4-hydroxy-3-methylbenzaldehyde (600 mg, 4.41 mmol), 1-(bromomethyl)-4-fluorobenzene (850 mg, 4.5 mmol), and potassium carbonate (1.22 g, 8.81 mmol) in DMF (12 mL) was stirred at 25 °C. After the reaction was judged to be completed by TLC (2 h), it was quenched with water and extracted with EtOAc/hexanes (*v*/*v* = 1:1). The organic layer was washed with water two times and brine and concentrated under reduced pressure by rotary evaporation. The crude residue was purified by flash column chromatography (SiO_2_: 0–25% EtOAc in hexanes) to give compound **1** as a yellow amorphous solid (1 g, 90%). ^1^H NMR (400 MHz, CDCl_3_) δ 9.87 (s, 1H), 7.74–7.67 (m, 2H), 7.41 (dd, *J* = 8.5, 5.4 Hz, 2H), 7.10 (t, *J* = 8.7 Hz, 2H), 6.97 (d, *J* = 8.2 Hz, 1H), 5.13 (s, 2H), 2.32 (s, 3H).

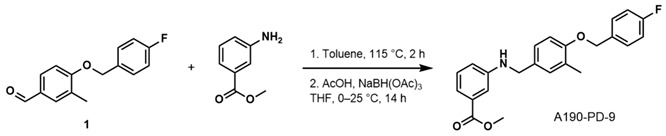

Methyl 3-((4-((4-Fluorobenzyl)oxy)-3-Methylbenzyl)amino)benzoate (A190-PD-9)

A mixture of **1** (244 mg, 1 mmol) and methyl 3-aminobenzoate (151 mg, 1 mmol) in toluene (5 mL) was stirred at 115 °C for 2 h. The solvent was removed under reduced pressure by rotary evaporation. The residue was dissolved in THF (5 mL) and cooled to 0 °C. Sodium triacetoxyborohydride (423 mg, 2 mmol) was added and the mixture was stirred at this temperature for 15 min. Acetic acid (1 µL) was added and the mixture was stirred at 25 °C. After the reaction was judged to be completed by TLC (14 h), it was quenched with water and extracted with EtOAc. The organic layer was washed with water and brine and concentrated under reduced pressure by rotary evaporation. The crude residue was purified by flash column chromatography (SiO_2_: 0–20% EtOAc in hexanes) to afford compound A190-PD-9 as a yellow amorphous solid (273 mg, 72%). ^1^H NMR (400 MHz, DMSO) δ 7.49 (dd, *J* = 8.5, 5.7 Hz, 2H), 7.29–7.05 (m, 7H), 6.95 (d, *J* = 8.3 Hz, 1H), 6.84–6.78 (m, 1H), 6.47 (t, *J* = 5.9 Hz, 1H), 5.06 (s, 2H), 4.18 (d, *J* = 5.9 Hz, 2H), 3.79 (s, 3H), 2.16 (s, 3H). ^13^C NMR (101 MHz, DMSO) δ 166.7, 161.6 (d, *J* = 243.4 Hz), 155.1, 148.9, 133.7 (d, *J* = 3.0 Hz), 131.4, 130.2, 129.6, 129.4 (d, *J* = 8.2 Hz), 129.1, 125.8, 125.7, 116.6, 116.3, 115.2 (d, *J* = 21.6 Hz), 112.6, 111.6, 68.5, 51.9, 45.8, 16.2. TOFMSESI *m*/*z*: 402.1468 (C_23_H_22_FNO_3_ + Na^+^ requires 402.1476).

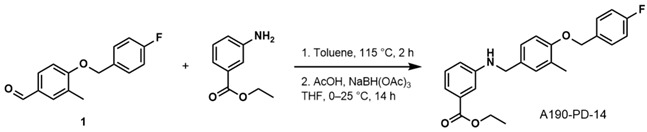

Ethyl 3-((4-((4-Fluorobenzyl)oxy)-3-Methylbenzyl)amino)benzoate (A190-PD-14)

A mixture of **1** (244 mg, 1 mmol) and ethyl 3-aminobenzoate (165 mg, 1 mmol) in toluene (5 mL) was stirred at 115 °C for 2 h. The solvent was removed under reduced pressure by rotary evaporation. The residue was dissolved in THF (5 mL) and cooled to 0 °C. Sodium triacetoxyborohydride (423 mg, 2 mmol) was dded and the mixture was stirred at this temperature for 15 min. Acetic acid (1 µL) was added and the mixture was stirred at 25 °C. After the reaction was judged to be completed by TLC (14 h), it was quenched with water and extracted with EtOAc. The organic layer was washed with water and brine and concentrated under reduced pressure by rotary evaporation. The crude residue was purified by flash column chromatography (SiO_2_: 0–20% EtOAc in hexanes). The appropriate fractions were collected and concentrated under reduced pressure by rotary evaporation; the residue was further purification by recrystallization in ethanol to afford compound A190-PD-14 as a white amorphous solid (142 mg, 36%). ^1^H NMR (400 MHz, DMSO) δ 7.49 (dd, *J* = 8.5, 5.7 Hz, 2H), 7.24–7.06 (m, 7H), 6.95 (d, *J* = 8.3 Hz, 1H), 6.83–6.75 (m, 1H), 6.48 (t, *J* = 5.9 Hz, 1H), 5.06 (s, 2H), 4.24 (q, *J* = 7.1 Hz, 2H), 4.17 (d, *J* = 5.9 Hz, 2H), 2.16 (s, 3H), 1.28 (t, *J* = 7.1 Hz, 3H). ^13^C NMR (101 MHz, DMSO) δ 166.2, 161.7 (d, *J* = 243.3 Hz), 155.1, 148.9, 133.7 (d, *J* = 3.0 Hz), 131.4, 130.4, 129.6, 129.5 (d, *J* = 8.4 Hz), 129.0, 125.8, 125.7, 116.5, 116.3, 115.2 (d, *J* = 21.3 Hz), 112.7, 111.6, 68.5, 60.4, 45.9, 16.2, 14.2. TOFMSESI *m*/*z*: 416.1664 (C_24_H_24_FNO_3_ + Na^+^ requires 416.1633).

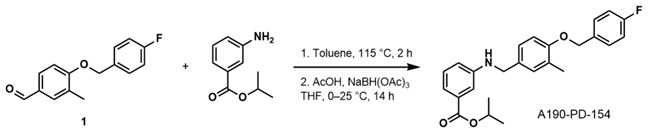

Isopropyl 3-((4-((4-Fluorobenzyl)oxy)-3-Methylbenzyl)amino)benzoate (A190-PD-154)

A mixture of **1** (206 mg, 0.843 mmol) and isopropyl 3-aminobenzoate (151 mg, 0.843 mmol) in toluene (5 mL) was stirred at 115 °C for 2 h. The solvent was removed under reduced pressure by rotary evaporation. The residue was dissolved in THF (5 mL) and cooled to 0 °C. Sodium triacetoxyborohydride (357 mg, 1.69 mmol) was added and the mixture was stirred at this temperature for 15 min. Acetic acid (1 µL) was added and the mixture was stirred at 25 °C. After the reaction was judged to be completed by TLC (14 h), it was quenched with water and extracted with EtOAc. The organic layer was washed with water and brine and concentrated under reduced pressure by rotary evaporation. The crude residue was purified by flash column chromatography (SiO_2_: 0–20% EtOAc in hexanes). The appropriate fractions were collected and concentrated under reduced pressure by rotary evaporation; the residue was further purification by recrystallization in isopropanol to afford compound A190-PD-154 as a white amorphous solid (100 mg, 29%). ^1^H NMR (400 MHz, DMSO) δ 7.49 (dd, *J* = 8.5, 5.7 Hz, 2H), 7.25–7.05 (m, 7H), 6.94 (d, *J* = 8.3 Hz, 1H), 6.80–6.75 (m, 1H), 6.48 (t, *J* = 5.9 Hz, 1H), 5.15–4.99 (m, 3H), 4.17 (d, *J* = 5.9 Hz, 2H), 2.16 (s, 3H), 1.28 (d, *J* = 6.2 Hz, 6H). ^13^C NMR (101 MHz, DMSO) δ 165.7, 161.6 (d, *J* = 243.3 Hz), 155.1, 148.8, 133.7 (d, *J* = 2.9 Hz), 131.4, 130.8, 129.6, 129.4 (d, *J* = 8.3 Hz), 129.0, 125.8, 125.7, 116.4, 116.3, 115.2 (d, *J* = 21.4 Hz), 112.8, 111.6, 68.5, 67.6, 45.9, 21.7, 16.2. TOFMSESI *m*/*z*: 430.1792 (C_25_H_26_FNO_3_ + Na^+^ requires 430.1789).

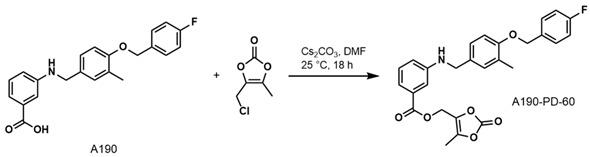

(5-Methyl-2-oxo-1,3-dioxol-4-yl)methyl3-((4-((4-Fluorobenzyl)oxy)-3-methylbenzyl) amino)benzoate (A190-PD-60)

To a solution of A190 (525 mg, 1.44 mmol) in DMF (57.5 mL) was added cesium carbonate (234 mg, 0.718 mmol). This mixture was allowed to stir for 15 min at 25 °C, then 4-(chloromethyl)-5-methyl-1,3-dioxol-2-one (213 mg, 1.44 mmol) was added in one portion. The mixture was stirred at 25 °C. After the reaction was judged to be completed by TLC (18 h), it was quenched with water and extracted with EtOAc. The organic layer was washed with water, concentrated sodium bicarbonate, and brine, then concentrated under reduced pressure by rotary evaporation. The crude residue was purified by flash column chromatography (SiO_2_: 0–40% EtOAc in hexanes) to give compound A190-PD-60 as an off-white amorphous solid (302 mg, 44%). ^1^H NMR (400 MHz, DMSO) δ 7.50 (dd, *J* = 8.5, 5.7 Hz, 2H), 7.27–7.09 (m, 7H), 6.95 (d, *J* = 8.3 Hz, 1H), 6.85–6.78 (m, 1H), 6.54 (t, *J* = 5.9 Hz, 1H), 5.16 (s, 2H), 5.07 (s, 2H), 4.18 (d, *J* = 5.9 Hz, 2H), 2.21 (s, 3H), 2.17 (s, 3H). ^13^C NMR (101 MHz, DMSO) δ 165.7, 161.7 (d, *J* = 243.3 Hz), 155.2, 151.9, 148.9, 140.1, 133.7 (d, *J* = 3.0 Hz), 133.5, 131.3, 129.5 (d, *J* = 11.9 Hz), 129.5, 129.4, 129.2, 125.8, 125.7, 116.9, 116.5, 115.2 (d, *J* = 21.4 Hz), 112.9, 111.6, 68.4, 54.2, 45.8, 16.2, 8.9. TOFMSESI *m*/*z*: 500.1450 (C_27_H_24_FNO_6_ + Na^+^ requires 500.1480).

### 2.2. Aqueous Solubility of Prodrugs

Aqueous solubility of each prodrug was assessed using deionized water. In brief, an excess amount of each prodrug was placed in a stoppered vial containing 1 mL of water. Each sample was vortexed and kept in an isothermal shaker maintained at 25 ± 1.0 °C to reach equilibrium for 48 h. The resulting mixture was centrifuged at 4000× *g* for 15 min, and the supernatant was collected and diluted with ACN followed by filtration with a 0.22 μm filter. The drug concentrations for all prodrugs were measured using HPLC-UV. Chromatographic separation was achieved using an Agilent Pursuit XRs 5 C18 column (250 × 4.6 mm) with a UV-detector system. The mobile phase consisted of ACN and water, both containing 0.1% trifluoroacetic acid in a ratio of 80:20 *v*/*v*, with a flow rate of 1 mL/min at 25 °C. Drugs were detected at a wavelength of 227 nm and different retention times were observed for each prodrug.

### 2.3. Bioconversion of Prodrugs in Plasma

Conversion of the respective prodrugs to the parent drug A190 was assessed in pooled rat and rabbit plasma. Following pre-incubation of plasma at 37 °C for 5 min, stock solution of each prodrug (10 mM) was spiked into the plasma to reach a final concentration of 100 μM. The mixture was incubated at 37 °C, 200 rpm on a temperature-controlled orbital shaker. Incubation mixture (200 μL) was withdrawn at pre-determined time points, and the reaction was terminated by the addition of 700 μL of ice cold ACN. 100 μL of internal standard (0.5 μg/mL of fenofibric acid) was then spiked, and the samples were vortexed and centrifuged at 10,000× *g* for 10 min. The resulting supernatants were collected and filtered using PTFE filters and injected into HPLC-UV system. The LC system was a reverse phased Shimadzu Prominence (Kyoto, Japan) using a Pursuit 5 C18 column. Isocratic separation was performed with mobile phase consisting of ACN/water (80/20 *v*/*v*) containing 0.1% trifluoroacetic acid and monitored at a UV detection of 227 nm.

### 2.4. Chemical Hydrolysis Stability of Prodrugs

Chemical hydrolysis stability of each prodrug was assessed in a physiological pH 7.4 using phosphate buffered saline solution (PBS) as previously described [25]. After preincubation of PBS solution at 37 °C for 5 min, stock solution of each prodrug (10 mM) was spiked to a final concentration of 100 μM. The samples were then incubated at 37 °C on a temperature-controlled orbital shaker operating at 200 rpm. At predetermined time points, 200 μL of incubation mixture was withdrawn and quenched with 100 μL of internal standard (0.5 μg/mL of fenofibric acid) and 700 μL ice cold ACN. Drug concentration was analyzed using HPLC-UV method as described earlier to assess the amount of prodrug recovered compared to the starting timepoint.

### 2.5. Preparation and Characterization of A190-PD-60 Microemulsion

#### 2.5.1. Solubility Based Selection of Microemulsion Components

Enhancement of drug solubility is the primary factor considered when evaluating ME components. To determine the solubility of A190-PD-60, an excess amount of A190-PD-60 was added to 1 g of each of the tested oils, surfactants and co-surfactants in glass vials with stoppers. A190-PD-60 and excipients were mixed together and vortexed. The mixtures were kept at a temperature of 25 ± 1.0 °C in an isothermal shaker for 72 h to achieve equilibrium. The mixtures were then centrifuged at 10,000× *g* for 15 min. The resulting supernatants were filtered using 0.45 mm membrane filters and concentrations of A190-PD-60 in the filtrates were quantified via HPLC-UV analysis. Oils, emulsifiers, and co-emulsifiers with the highest solubilities were chosen for further miscibility analysis. For this, 2 mL of the selected excipients were mixed and vortexed for 10 min, then allowed to equilibrate for 1 h. The resulting mixtures were visually inspected for transparency and phase separation.

#### 2.5.2. Formulation Optimization with Box-Behnken Experimental Design

A three-factor and three-level Box-Behnken design (BBD) was used for constructing the models of A190-PD-60 microemulsion (A190-PD-60-ME) as generated by JMP Pro 17 software (SAS Institute, Cary, NC, USA). BBD required 15 experimental runs with 3 central points to determine the experimental error and predict precision of the design. Independent variables selected for the study were percentage of oil (X_1_), percentage of surfactant (X_2_), and percentage of co-surfactant (X_3_). These independent variables were adjusted at three different levels: −1 (lower level), 0 (medium level), and +1 (higher level). Mean droplet size (Y_1_), polydispersity index (PDI) (Y_2_) and drug content (Y_3_) were considered as the response variables (Table 1). Statistical models were selected using significant highest-order polynomials while avoiding aliasing. The A190-PD-60-ME was optimized within the design space using predictor profiles to achieve optimal characteristics: minimal droplet size (<200 nm) and PDI (<0.3), maximal drug loading (>95%), and near-neutral zeta potential (−5 to +5 mV).

#### 2.5.3. Formulation of A190-PD-60-ME

A190-PD-60-MEs were formulated using different combinations of oil (7.5–22.5%), surfactant (5–20%), co-surfactant (5–20%) and deionized water (45–77.5%). Briefly, A190-PD-60 was first dissolved in Capryol 90 (oil) under sonication, followed by the addition of Tween 80 (surfactant) and Transcutol (co-surfactant). The mixture was further sonicated in a water bath for 30 min to ensure the drug was completely dissolved in the oil phase. After complete drug solubilization, deionized water was added into the oil phase under continuous stirring at the desired final concentration (5 mg/mL). Physicochemical characterization of A190-PD-60-MEs was performed using dynamic laser light scattering Malvern Zetasizer (Nano ZS90, Malvern Instruments Ltd., Worcestershir, UK) for mean droplet size, PDI, and zeta potential. Samples were prepared by diluting the microemulsion formulations with 10 mM NaCl (1:100 *v*/*v*), followed by 1 min sonication to reduce multiple scattering effects. All measurements were conducted at 25 °C in triplicate and reported as mean ± SD. Drug content analysis was performed by diluting formulations with ACN, filtering through a 0.22 µm membrane, and analyzed using HPLC-UV.

### 2.6. In-Vitro Permeability Assay

Parallel artificial membrane permeability assay (PAMPA) is a widely used screening method to predict the passive intestinal permeability using a phospholipid coated lipophilic membrane which simulates the properties of the intestinal wall [26]. The in vitro intestinal membrane permeability of free A190-PD-60 (dispersed in PBS pH 6.8), A190-PD-60 dispersion (dispersed in a mixture of 1:1 *v*/*v* methylcellulose/Tween 80), and A190-PD-60-ME were evaluated using a PAMPA plate (BD BioSciences, San Jose, CA, USA), as described previously [16]. The samples for donor chamber were prepared by diluting free A190-PD-60, A190-PD-60 dispersion, and A190-PD-60-ME with PBS pH 6.8 to a final concentration of 200 µg/mL. The acceptor plate was loaded with 0.3 mL of pH 6.8 PBS, then sandwiched with the donor plate, ensuring that the membrane of the donor plate was in proper contact with the media in the acceptor plates. Next, 0.2 mL of the diluted samples were loaded in each well of the donor plate. The plate was then incubated at room temperature for 5 h. After 5 h, samples were withdrawn from both donor and acceptor chambers. The concentration of A190-PD-60 which permeated into the acceptor chamber was measured using a HPLC-UV system. The effective permeability coefficient (Pe) was then calculated using the following equation:

P_e_ = −ln(1 − C_A_[t]/C_equilibrium_)/(A × [1/V_D_ + 1/V_A_] × t) (1)

where P_e_ is the permeability (cm/s), A is the effective filter area (0.228 cm^2^), V_D_ is the volume of the donor well (0.2 mL), V_A_ is the volume of the receptor well (0.3 mL), t is the total time of incubation in seconds, C_A_(t) denotes the concentration of drug in the receptor well at time t, and C_equilibrium_ represents (C_D_[t] × V_D_ + C_A_[t] × V_A_)/(V_D_ + V_A_), where C_D_(t) denotes the concentration of drug in the donor well at time t.

### 2.7. Cell Viability Studies

To evaluate the cytotoxicity of free A190-PD-60 (in 0.2% DMSO in DMEM), vehicle microemulsion, and A190-PD-60-ME against Caco-2 cells and HepG2 cells, an MTT assay using 3-(4,5-dimethylthiazol-2-yl)-2,5-diphenyltetrazolium bromide was performed as described before [16]. Caco-2 and HepG2 cells were first cultured in DMEM supplemented with 10% FBS, penicillin (100 U/mL), and streptomycin (100 μg/mL) at 37 °C in a 5% CO_2_ humidified incubator. Upon reaching confluence, cells were trypsinized and seeded at 10 × 10^4^ cells/well in 96-well plates. After 48 h, the medium was replaced with fresh dilutions of free A190-PD-60, vehicle microemulsion, or A190-PD-60-ME (0.1–50 µM) in DMEM. Controls included untreated cells and cells treated with 0.2% DMSO in DMEM. Following 24 h and 48 h incubation, the medium was replaced with 100 µL of MTT solution (5 mg/mL in PBS) and incubated for 3 h. The supernatant was then removed, and formazan crystals were dissolved in 100 µL DMSO. Absorbance was measured at 540 nm, and cell viability (%) was calculated as (OD of samples/OD of control) × 100.

### 2.8. Stability Studies

To assess its storage stability, A190-PD-60-ME was stored in glass scintillation vials and kept at room temperature (25 ± 5 °C) for 3 months. At predetermined timepoints, droplet size, PDI, zeta potential, and percentage drug content were evaluated. Visual inspections for any signs of phase separation, turbidity, precipitation or color change were also performed for the duration of storage. Each experiment was performed in triplicate, and results were presented as mean ± SD.

### 2.9. Animals

The animal experimental protocol was approved by the Institutional Animal Care and Use Committee (IACUC) of Virginia Commonwealth University. Male Sprague Dawley (SD) rats (8-weeks old) were obtained from Envigo (Indianapolis, IN, USA). The experimental animals were cared for by the VCU’s Department of Animal Resources. All animal experiments were performed in accordance with the NIH Guidelines regarding the Care and Use of Laboratory Animals and the guidelines of the IACUC.

### 2.10. In-Vivo Oral Pharmacokinetics of A190-PD-60-ME

The absorption of A190-PD-60 from A190-PD-60-ME was evaluated following oral administration of the microemulsion to rats. Each rat was given an oral gavage of A190-PD-60-ME (20 mg/kg) or A190-PD-60 dispersion in a mixture of 1:1 *v*/*v* methylcellulose/tween80 (20 mg/kg). Post administration, 200 μL blood samples were collected using a heparinized capillary tube via tail vein at predetermined time intervals, and immediately centrifuged (2500× *g*, 15 min, 4 °C). The plasma was separated and kept frozen at −80 °C until analysis. To measure drug concentration in the plasma, drug was extracted using liquid phase extraction method and quantified using a validated LCMS method. Briefly, 150 μL of plasma sample was mixed with 100 μL of standards and 100 μL of internal standard (0.5 μg/mL fenofibric acid) and vortexed for 5 min. The plasma samples were extracted using 600 μL of ice cold ACN followed by vortex for 10 min. Then the samples were centrifuged at 10,000× *g* for 10 min, and the organic phase was collected in glass vials. The organic phase was then evaporated under nitrogen gas at 40 °C. Dry residues were reconstituted with 150 μL of mobile phase (1:1 ratio of ACN: Water with 0.1% formic acid). The reconstituted samples were well vortexed for 20 min and centrifuged (10,000× *g* for 10 min). A190-PD-60 and internal standard were separated using Phenomenex LC column (Luna Omega 3 μm Polar C18 100 Å, with dimensions of 100 mm × 2.1 mm). The mobile phase composition was ACN: H_2_O (60:40 *v*/*v*) containing 0.1% formic acid (flow rate 0.4 mL/min). Electron spray ionization (ESI) interface with positive ionization was used. The optimized conditions for LCMS were-interface temperature, 350 °C; DL temperature, 250 °C; nebulizing gas flow, 1.5 L/min; drying gas flow, 12 L/min. Pharmacokinetic parameters were estimated using a noncompartmental method using pK solver software [27].

### 2.11. Statistical Analysis

All statistical analyses were completed using GraphPad Prism 10.1, GraphPad Software Inc., San Diego, CA, USA). Statistical evaluation was done using student’s t-test for comparing two experimental groups and one-way or two-way ANOVA for comparing three or more groups. Differences were considered statistically significant if *p* < 0.05.

## 3. Results

### 3.1. Prodrugs Plasma Bioconversion

**Figure 3:**
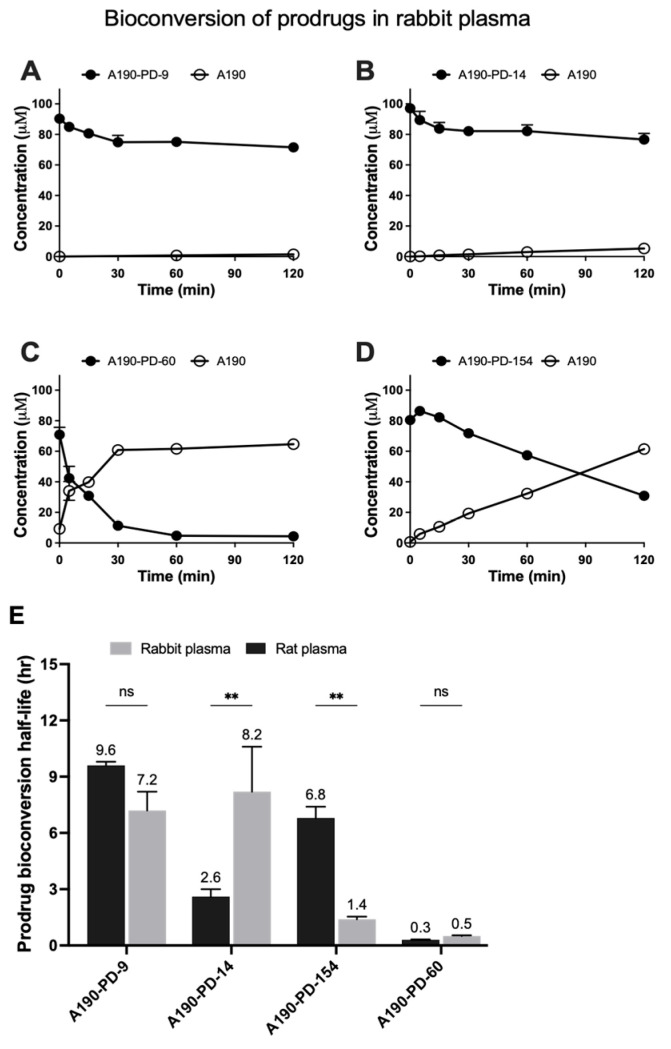
Bioconversion profile of A190 prodrugs. Concentration-time bioconversion profiles for (**A**) A190-PD-9, (**B**) A190-PD-14, (**C**) A190-PD-60, and (**D**) A190-PD-154 into parent drug A190 in rabbit plasma. Each prodrug (100 μM) was incubated with rabbit plasma at 37 °C for 120 min. (**E**) Bioconversion half-lives (t_1/2_) of prodrugs in rat and rabbit plasma. Data represented as Mean ± SD; n = 3, ** *p* < 0.01, ns: non-significant.

An ideal prodrug should be stable yet readily converted to the parent drug under enzymatic hydrolysis. The bioconversion profiles in rabbit (Figure 3A–D) and rat plasma (Figure S5A–D) demonstrate distinct stability and cleavage patterns between the four prodrugs. A190-PD-60 showed efficient cleavage in both rat and rabbit plasma, with prodrug levels dropping sharply within 120 min and a corresponding rise in A190 levels. The short half-lives (0.3 h in rat, 0.5 h in rabbit; panel E) indicate strong esterase recognition and prodrug cleavage efficiency. In contrast, A190-PD-9 exhibited minimal hydrolysis, retaining ≥80% of its original concentration after 120 min in both species, with longer half-lives (9.6 h in rat, 7.2 h in rabbit) and limited A190 release, raising potential concerns on insufficient in-vivo conversion of prodrug. A190-PD-14 and A190-PD-154 demonstrated species-specific hydrolysis behavior. A190-PD-14 was cleaved significantly faster in rats (2.6 h) than rabbits (8.2 h; *p* < 0.01), while A190-PD-154 showed the opposite trend (1.4 h in rabbit vs. 6.8 h in rat; *p* < 0.01). These results suggest species-dependent esterase interactions affecting the cleavage of ester-based prodrugs [28,29], suggesting potential challenges in translational consistency. Overall, A190-PD-60 emerged as the most promising candidate, combining favorable stability with efficient release of the parent drug in two different species.

### 3.2. Chemical Hydrolysis of Prodrugs

**Figure 4:**
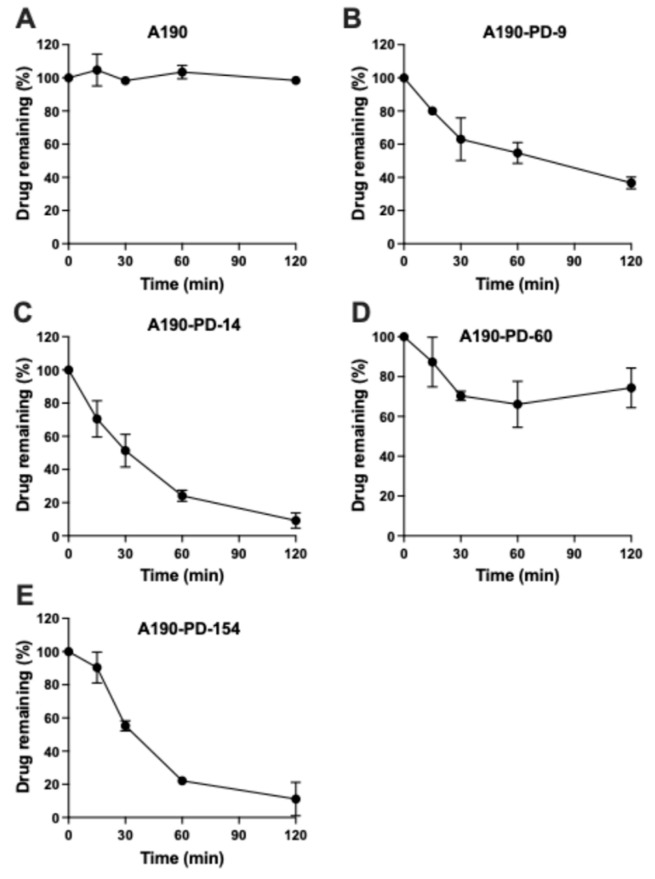
Chemical hydrolysis stability of A190 prodrugs. Each compound (**A**) A190, (**B**) A190-PD-9, (**C**) A190-PD-14, (**D**) A190-PD-60, and (**E**) A190-PD-154 was incubated with phosphate buffered saline (PBS) at 37 °C at a final concentration of 100 µM. The percentage (%) of drug remaining at predetermined time points till 120 min was quantified using HPLC analysis. Data represented as Mean ± SD; n = 3.

Each prodrug exhibited significant differences in their stabilities under aqueous conditions, which was evaluated in presence of phosphate buffered saline (PBS) (pH 7.4) at 37 °C (Figure 4). A190-PD-14 and A190-PD-154 exhibited the fastest degradation, with 10% of drug levels remaining at 2 h. A190-PD-9 showed moderate stability, retaining about 40% of the drug at 2 h. In contrast, A190-PD-60 displayed excellent chemical stability, with over 80% of the drug remaining at 2 h. The exceptional stability of A190-PD-60 could be attributed to its cyclic ester structure, which likely provides steric hindrance, and conformational nuances that make it more resistant to hydrolytic degradation [18]. Owing to its superior performance in enzymatic conversion and chemical stability, A190-PD-60 was selected for further formulation development and in-vivo evaluation.

### 3.3. Formulation Optimization of A190-PD-60-ME

#### 3.3.1. Solubility Analysis of Microemulsion Components

As shown in Table 2, the solubility study of A190-PD-60 was conducted using various excipients (oil, surfactants, co-surfactants) to optimize the stability of microemulsion. Surfactant and cosurfactant that demonstrated highest solubilities of prodrug with appropriate miscibility and proven safety were considered. Capryol 90 (15% *v*/*v*) (4.45 ± 0.31 mg/mL) was selected as the oil component in the formulation, as it showed much higher prodrug solubility than oleic acid and Maisine oil (4.8 and 13.9-fold, respectively). Tween 80 (Polysorbate 80) (20% *v*/*v*), a widely used nonionic surfactant, was selected as the surfactant based on its ability to dissolve A190-PD-60 at 9.17 ± 0.07 mg/mL. The hydrophobic carbon chains and hydrophilic ether groups of tween 80 makes it an excellent candidate for emulsification applications across the cosmetic, pharmaceutical, and food industries [30]. Transcutol HP (20% *v*/*v*) was chosen as the co-surfactant, showing very high solubility of the prodrug and good miscibility with Tween 80 compared to the other surfactants. Co-surfactants aid in further reducing the interfacial tension and provides flexibility to the surfactant film, enhancing the stability and reducing the droplet size of the microemulsion.

#### 3.3.2. Box-Behnken Experimental Design

A Box-Behnken design (BBD) was conducted using three factors at three levels each, following preliminary screening tests which identified the oil, surfactant, and co-surfactant candidates. Experimental conditions, parameters and desired outcomes of the BBD are listed in Table 1. The experimental data underwent analysis using multiple linear regression, specifically employing a quadratic fit. This analysis was carried out using JMP Pro 17 software, which was used to identify the polynomial model that best fit the data. Results from the experimental design, including the independent variables and responses for each experimental run can be seen in Table 3. Across 15 experimental runs, the droplet size ranged between 118.8 ± 1.38 to 1114.0 ± 69.5 nm, PDI between 0.26 ± 0.20 and 0.96 ± 0.05, and zeta potentials between −1.52 ± 0.3 and −4.2 ± 1.3 mV. Drug content between runs also showed some fluctuation, ranging from as low as 70 to as high as 104%. All 15 suggested formulations and their visual stabilities at room temperature are presented in Figure S6.

Table 4 summarizes the key statistical measures for the multiple linear regression model fit using this data, including *p*-values from the Analysis of Variance (ANOVA) analysis. These values collectively provide insight into the model’s performance and adequacy. It was observed that the percentages of Capryol 90 (*p* = 0.025) and Tween 80 (*p* = 0.0072) added to the formulations were critical in influencing the droplet size, while Tween 80 also had a critical impact on zeta potential (*p* = 0.047). Finally, the percentage of Transcutol P was found to significantly influence the drug content (*p* = 0.0495), but all other relationships were found to be not statistically significant. 3D surface plots illustrating the relationships between Capryol 90 (%), Tween 80 (%), Transcutol HP (%) and various dependent variables are presented in Figure 4. As Tween 80 percentage increased (moving along its axis), the droplet size decreased. As Capryol 90 percentage increased, the droplet size tended to increase. The effect of Transcutol HP in this plot appears to be moderate. As the percentage of Transcutol HP increased, there is a slight increase in droplet size, but the effect is less pronounced compared to Capryol 90.

**Figure 5:**
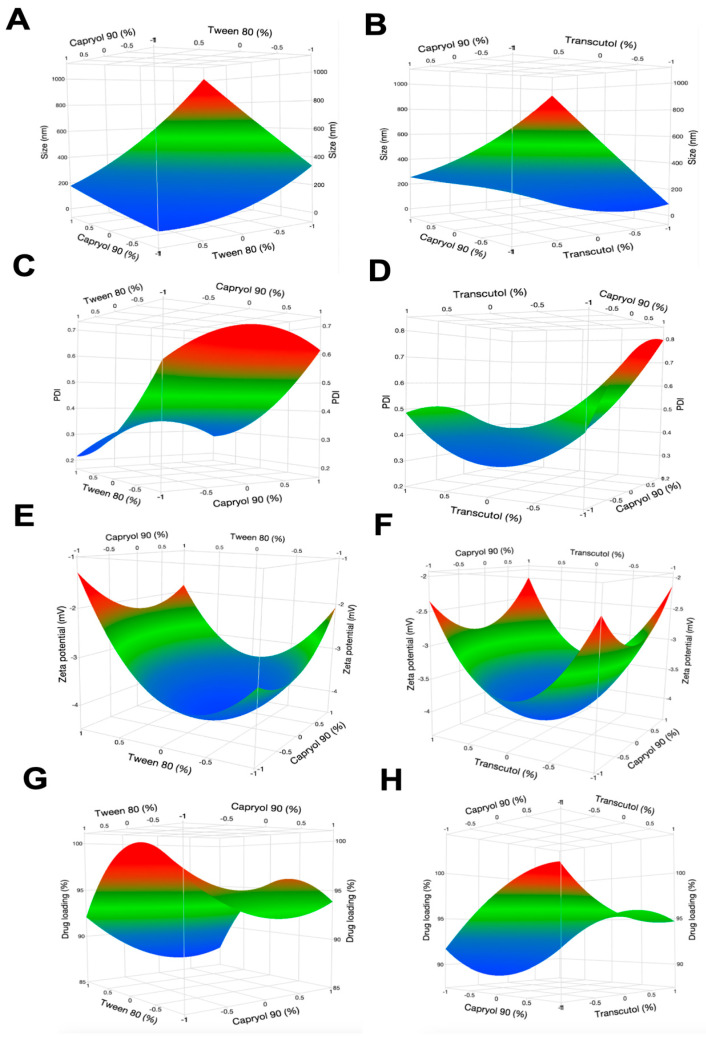
3D response surface plots as generated by BBD representing the effect of independent variables on dependent variables. (**A**,**C**,**E**,**G**) Effect of Oil (% Capryol 90) and Surfactant (% Tween 80) on size, PDI, zeta potential and drug content. (**B**,**D**,**F**,**H**) Effect of Oil (% Capryol 90) and Co-surfactant (% Transcutol HP) on size, PDI, zeta potential and drug content.

Based on the surface profiler diagram (Figure 5), lower PDI values were prominent in the areas with higher percentages of Tween 80 and lower percentages of Capryol 90 and Transcutol HP. This suggests that increasing the proportion of Tween 80 in the formulation tended to result in a lower PDI and more uniform droplet size distribution. In contrast, the PDI appears to be less sensitive to changes in Capryol 90 and Transcutol HP percentages, as evidenced by the relatively smaller variation in color along these axes. This observation is consistent with the marginal *p*-value of 0.06 for Tween 80 composition, indicating its stronger influence on PDI compared to the other two components. It was also observed that increasing %Transcutol HP can positively impact drug loading. Capryol 90 has a very minimal effect on drug loading with no obvious trends observed. The results suggest that focusing on optimizing Transcutol concentration, while carefully balancing tween 80 levels, could be critical to improve drug loading in this microemulsion system.

The relationship between tween 80 concentration and zeta potential is nonlinear. At low-to-medium concentrations, increasing tween 80 intensifies the negative charge, but this trend reverses at higher concentrations, where zeta potential becomes less negative. This curvature, supported by statistical significance (*p* = 0.047), highlights Tween 80′s complex influence. In contrast, Capryol 90 (oil phase) exerts a milder effect, with higher concentrations slightly reducing the negative charge, particularly when Tween 80 levels are low. Transcutol, despite its pronounced impact on drug loading efficiency, shows negligible influence on zeta potential, emphasizing how different excipients can selectively affect microemulsion properties. Based on its desirable physicochemical properties, excellent long term stability (no phase separation, precipitation, discoloration), and close resemblance with maximum desired formulation suggested by Box-Behnken analysis, formulation #8 (hereafter named as A190-PD-60-ME) was selected for further analysis.

### 3.4. In-Vitro Parallel Artificial Membrane Permeability

Analysis of passive diffusion of free A190-PD-60, A190-PD-60 dispersion, and A190-PD-60-ME through artificial intestinal membrane was performed in vitro, using artificial phospholipid membrane. It was observed that the permeation of A190-PD-60 dispersion through the membrane was significantly lower (Pe = 0.1 × 10^−6^ cm/s) than A190-PD-60-ME (Pe = 10.8 × 10^−6^ cm/s) (Table 5), suggesting its potential for enhanced permeability when delivered in-vivo. For comparison, previously reported permeability data for the parent drug A190 showed that A190 in PBS had low permeability (Pₑ = 0.09 ± 0.02 × 10^−6^ cm/s), which increased slightly in dispersion form (Pₑ = 0.13 ± 0.04 × 10^−6^ cm/s), and was further improved with microemulsion formulation (A190-ME, Pₑ = 1.16 ± 0.20 × 10^−6^ cm/s) [16]. It highlights the ability of microemulsions to markedly enhance membrane permeability of both the parent drug and its prodrug, supporting their application in improving oral drug delivery.

Surfactant and cosurfactants used in microemulsion formulations improve drug permeability via various mechanisms. Essentially, these agents improve the solubility of poorly water-soluble drugs such as A190-PD-60 by generating micellar structures, which increases drug solubility in the aqueous phase [31]. Second, greater concentrations of surfactants and co-surfactants change the manner in which the drug partitions between the oil and water phases of the microemulsion, increasing drug loading in the oil phase and encouraging passive diffusion across biological membranes [32]. They also minimize interfacial tension between the oil and aqueous phases, which stabilizes the microemulsion structure and allows for closer contact with the membrane surface, resulting in better drug release [33]. Finally, some surfactants can interact with membrane lipids, increasing the membrane fluidity and permeability of drugs [34,35].

### 3.5. Cell Cytotoxicity Analysis

**Figure 6:**
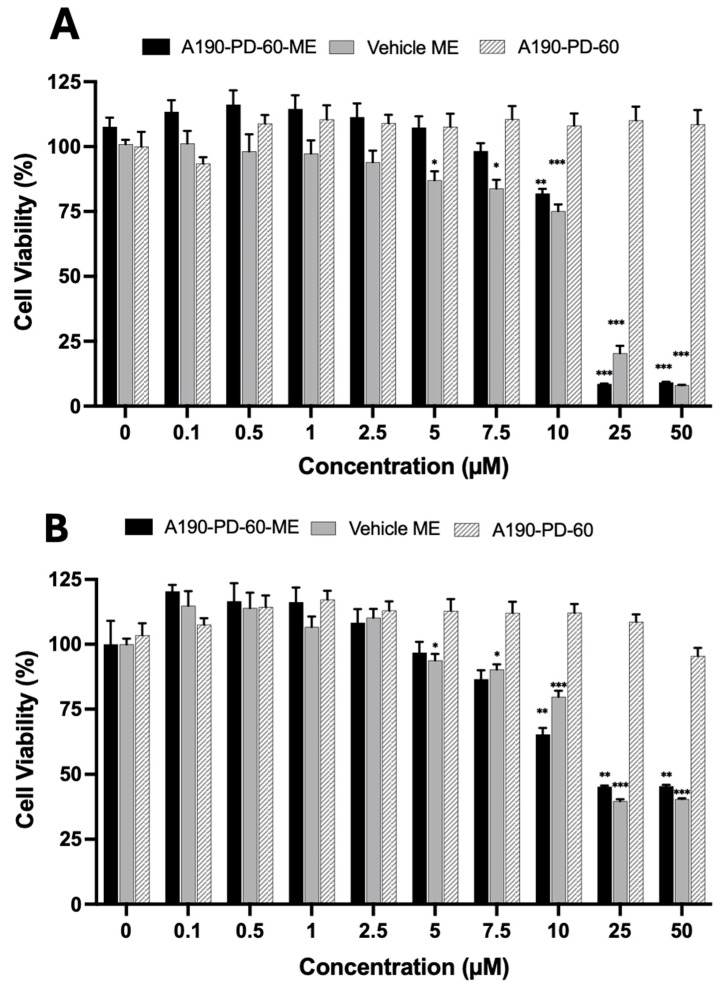
In-vitro cell cytotoxicity assay of A190-PD-60 (in 0.2% DMSO), vehicle microemulsion, and A190-PD-60-ME on (**A**) HepG2 cells and (**B**) Caco-2 cells after incubation for 24 h. Data is represented as Mean ± SEM; n = 4–5. * *p* < 0.05, ** *p* < 0.01, *** *p* < 0.001 compared to respective DMEM or 0.2% DMSO controls.

To evaluate cytotoxicity, an MTT assay was performed on Caco-2 and HepG2 cell lines using free A190-PD-60 (dissolved in 0.2% DMSO), the vehicle microemulsion (without drug), and A190-PD-60-ME (drug-loaded microemulsion). Free A190-PD-60 demonstrated no significant cytotoxicity, even at the highest tested concentration (50 µM), in both cell lines after 24 h and 48 h of exposure. The vehicle microemulsion alone showed high biocompatibility, maintaining cell viability above 80% at concentrations ≤ 7.5 µM, which confirms the safety of the formulation components. Similarly, the drug-loaded A190-PD-60-ME exhibited no toxicity at concentrations ≤ 7.5 µM, with cell viability remaining >80% (Figure 6). However, at higher concentrations, a concentration-dependent decrease in cell viability was observed for A190-PD-60-ME in both cell lines. As previously reported, parent compound A190 (in 0.2% DMSO) was also safe up to a concentration of <50 µM in both cell lines at 24 h and 48 h [16]. These results are consistent with previous findings on the toxicity of microemulsions composed of similar components in cell-based studies [36].

### 3.6. Stability of A190-PD-60-ME

**Figure 7:**
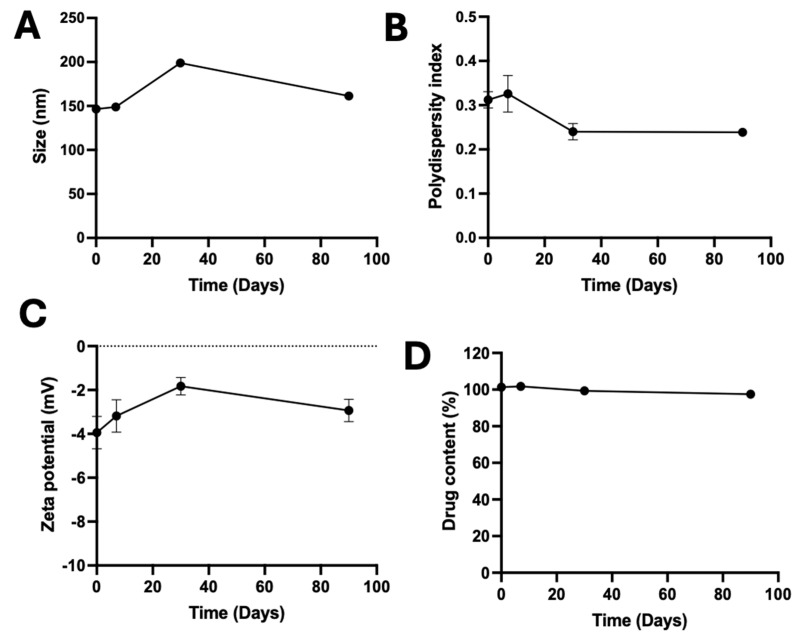
Storage stability of A190-PD-60-ME at room temperature (25 ± 5 °C). (**A**) Average droplet size, (**B**) Polydispersity index, (**C**) Zeta potential, and (**D**) Drug content of A190-PD-60-ME. Data is represented as Mean ± SD; n = 3.

No phase separation, precipitation, creaming was observed during the study period, which confirmed the formation of a thermodynamically stable microemulsion. In a long-term storage stability analysis of A190-PD-60-ME at room temperature (25 ± 5 °C), the drug content was maintained at >95% with no significant changes in the mean droplet size, PDI or zeta potential over time (Figure 7).

### 3.7. In-Vivo Oral Pharmacokinetic Study

**Figure 8:**
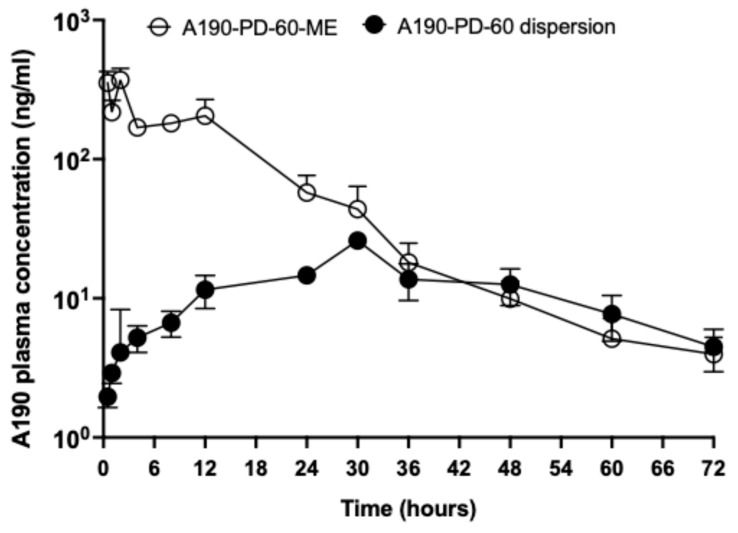
Mean plasma concentration-time profiles of A190 after oral administration of A190-PD-60 dispersion (20 mg/kg) and A190-PD-60-ME (20 mg/kg) to rats. A190-PD-60 dispersion was prepared in the mixture of 1:1 *v*/*v* methylcellulose/tween80 in autoclaved water. Data represent Mean ± SEM; n = 4–5.

The plasma concentration (of parent drug A190) time profiles and the pharmacokinetic parameters of A190-PD-60-ME and A190-PD-60 dispersion in rats are presented in Figure 8 and Table 6 respectively. The intestinal absorption was improved significantly with the microemulsion formulation compared to the dispersion. The AUC of A190-PD-60-ME was 4914.33 ± 525.79 ng/mL × h, which was significantly higher (5.3-fold) compared to A190-PD-60 dispersion (828.10 ± 102.86 ng/mL × h). The maximum concentration (C_max)_ for A190-PD-60-ME was 438.85 ± 74.55 ng/mL, which was 16.6-fold higher than A190-PD-60 dispersion (26.47 ± 0.55 ng/mL). It was observed that the time to reach maximum concentration (T_max_) was relatively short (1.7 ± 0.3 h) for the microemulsion formulation compared to the A190-PD-60 dispersion (31.5 ± 1.5 h). The concentration-time profile of A190-PD-60-ME exhibited a biphasic elimination pattern, with a sharp initial decline followed by a more gradual decrease. In contrast, the A190-PD-60 dispersion maintained a relatively constant, low plasma concentration throughout the 72-h observation period. These distinct profiles suggest that the microemulsion formulation may offer advantages in terms of faster onset of action and potentially greater overall drug exposure, as indicated by the larger area under the curve. The half-lives (t_1/2_) observed for the A190-PD-60-ME and A190-PD-60 dispersion were 9.32 ± 1.27 h and 14.11 ± 1.94 h, respectively. In addition, the relative oral bioavailability of A190-PD-60-ME was 5.93-fold higher compared to A190-PD-60 dispersion.

## 4. Discussion

In this study, we compared plasma-mediated cleavage of four A190 prodrugs (PD-9, PD-14, PD-60, PD-154). An ideal prodrug should resist nonspecific degradation during circulation yet still undergo enzymatic conversion to the parent drug [37,38]. Of the four different candidates, A190-PD-9 and A190-PD-14 were stable in plasma matrix but exhibited a very slow conversion to A190. In comparison, A190-PD-60 and A190-PD-154 showed much improved concentration-time profiles. However, A190-PD-154 released A190 with a prolonged lag, whereas A190-PD-60 generated an immediate surge of parent drug, yielding the most favorable concentration-time profile for application in oral delivery.

In preparation for microemulsion, a solubility study of A190-PD-60 in an array of oils, surfactants and cosurfactants was performed. Compared to Oleic acid and Maisine oil, the A190-PD-60 exhibited much higher solubility in Capryol 90. Capryol 90, which is composed of propylene glycol esters of caprylic acid, has also exhibited superior solubilizing capacity and low levels of toxicity for human intake in other studies [39], which made it an appropriate choice for the oil component in this formulation. In terms of surfactant selection, Tween 80 was chosen, as it showed high solubility compared to other components tested. Tween 80 is widely used in other reported oral microemulsion formulations and shows many advantages. The hydrophobic carbon chains and hydrophilic ether groups of Tween 80 make it an excellent candidate for emulsification applications across the cosmetic, pharmaceutical, and food industries [30]. Similarly, Tween 80 has minimal sensitivity to variations in pH and ionic strength. The co-surfactant (Transcutol HP in this case) is selected based on its ability to lower the interfacial tension between the oil and water phases, ensuring the formation of a stable microemulsion [40]. A190-PD-60 showed extremely high solubility in Transcutol HP compared to its competitors (propylene glycol, Cremophor EL, Peceol). In addition to the interfacial tension reduction, the cosurfactant provides flexibility to the surfactant film enhancing the stability and reducing the droplet size of the microemulsion.

Previous studies have demonstrated that incorporation of Transcutol HP in microemulsion system promoted solubilization, stability and intestinal permeability [41,42]. While certain components like Cremophor EL provided great solubility of A190-PD-60 (9.05 ± 0.04 mg/mL), we excluded them from the final lead formulation considering the potential concerns of hypersensitivity with its use [43].

Through the Box Behnken Experimental Design, we were able to evaluate the effects that these chosen excipients had on critical quality attributes of the microemulsion formulation—droplet size, zeta potential, and percent drug loading. Primarily, droplet size is a crucial parameter for microemulsion formulations. Previous studies have reported that smaller droplets are less likely to coalesce or undergo Ostwald ripening, leading to improved long-term stability of microemulsion [44]. Smaller droplet sizes can also increase the surface area-to-volume ratio, which enhances drug dissolution and absorption—potentially leading to improved bioavailability of poorly water-soluble drugs [45]. Because of this, it is important for our microemulsion formulation to have a minimized droplet size, which typically means less than about 100 nm.

PDI is another important characteristic which quantifies the uniformity and size distribution of particles in a formulation. A lower PDI (typically < 0.3) indicates a more homogeneous size distribution, which is desirable for consistent performance and stability of nanoparticle-based drug delivery systems [46]. The results of this BBD also revealed that none of the factors significantly impacted the PDI response of the formulations, though Tween 80 (surfactant) composition did seem to trend towards having an impact on it. The amount of Capryol 90 and Transcutol HP in the formulations did not seem to have much influence on the uniformity of the emulsions.

Zeta potential, or surface charge, can also be an indicator of the stability of microemulsions, as a higher absolute value typically is correlated to enhanced stability through the promotion of electrostatic repulsion between droplets [47]. The observed zeta potential range (−1.5 to −4 mV) in this system suggests that electrostatic forces are likely not the dominant stabilization mechanism. Instead, steric stabilization from the nonionic surfactant Tween 80 appears critical, forming a protective barrier around droplets to prevent aggregation. In these fifteen formulations tested, it seems that the only factor which significantly impacted the zeta potential was the amount of Tween 80 (surfactant).

Drug loading of microemulsion is critical to its performance in-vivo. The results show that the amount of cosurfactant added to the formulation does significantly impact the drug content, while the other factors do not. Tween 80 is observed to have both positive and negative impact on drug loading. In previous studies, it was shown that moderate amounts of Tween 80 promoted drug loading and entrapment efficiency, while higher or lower amounts led to aggregation and instability, which in turn hindered the drug loading [48]. Alternatively, the amount of Capryol 90 seems to have a very minimal effect on drug loading, with no obvious trends observed. Overall, the results from this DOE highlight the complex interplay between excipients in microemulsion formulations and underscore the importance of optimizing each component’s concentration to achieve maximum drug loading. When evaluated for stability up to 3 months, A190-PD-60-ME exhibited excellent stability, confirming its thermodynamically stable nature and viability of use after long-term storage.

When tested in rats, A190-PD-60-ME exhibited improved pK performance as compared to the A190-PD-60 dispersion. In previous studies, oral microemulsions have been shown to bypass the hepatic portal route, promote lymphatic transport of lipophilic drugs, reduce cytochrome P450 metabolism in enterocytes, and overcome enterohepatic recirculation [49], which would explain the observed increase in drug exposure. Moreover, the enhanced oral absorption and bioavailability of A190-PD-60-ME could be attributed to several mechanisms such as paracellular transport where surfactants in the microemulsion may open tight junctions between intestinal epithelial cells, allowing for increased paracellular transport of the drug [50]. Similarly, the oil components in the microemulsion are likely digested by pancreatic lipases, creating an environment where A190-PD-60 remains solubilized [51,52]. This solubilized drug can then passively diffuse across the intestinal epithelium more efficiently than the suspension form, explaining the higher overall absorption. In contrast, the suspension may precipitate, limiting its absorption, as evidenced by its lower and slower absorption profile. The drug particles in the suspension dissolve gradually in the gastrointestinal tract, leading to prolonged, rate-limited absorption, a characteristic of flip-flop kinetics [53].

Based on our current study, A190-PD-60 exhibits an aqueous solubility of 1.4 ± 0.23 µg/mL and a PAMPA permeability (Pe) of <0.1 × 10^−6^ cm/s. Given its low solubility and low permeability, A190-PD-60 is predicted to fall under BCS Class IV [54]. This classification highlights the challenge of achieving sufficient oral bioavailability and justifies our strategy of utilizing both prodrug design and a microemulsion-based delivery system to enhance solubility and promote GI absorption.

## 5. Conclusions and Future Directions

This study explored the synthesis of four novel A190 prodrugs aimed for oral delivery of non-fibrate PPARα agonists. Among them, A190-PD-60 emerged as the lead candidate based on its favorable chemical stability and efficient plasma cleavage. To further enhance its performance, A190-PD-60 was formulated into an oral microemulsion (A190-PD-60-ME). A190-PD-60-ME achieved a 5.9-fold increase in the oral bioavailability of A190 compared to its dispersion form in an oral pharmacokinetic study. These findings highlight the synergistic potential of combining prodrug strategy with microemulsion-based delivery to overcome solubility and permeability limitations of A190 and enhance its oral bioavailability. Future efforts should focus on further optimizing prodrug chemistry (e.g., activated esters, steric modifications) in order to enhance hydrolysis efficiency. Similarly, leveraging alternative oral formulation strategies such as solid self-emulsifying formulations for sustained release, and ultimately testing therapeutic outcomes of these delivery systems in PPARα associated disease models would be crucial in the future. Furthermore, we employed the PAMPA-based permeability model in the current study. Future investigations shall incorporate Caco-2-based permeability assays to account for the potential contribution of active transport and efflux mechanisms.

## Figures and Tables

**Table 1:** Dependent and independent variables for Box-Behnken experimental design.

Independent Variables	Levels		
	Low (−1)	Medium (0)	High (+1)
Factor 1 (X_1_): Oil (%)	7.5	15	22.5
Factor 2 (X_2_): Surfactant (%)	5	10	20
Factor 3 (X_3_): Co-surfactant (%)	5	10	20
Dependent variables	Constraints		
Response 1 (Y_1_): Mean droplet size	Minimum		
Response 2 (Y_2_): Polydispersity index	Minimum		
Response 3 (Y_3_): Zeta potential	In range		
Response 4 (Y_4_): Drug content	Maximum		

**Table 2:** Solubilities of A190-PD-60 in different excipients.

Excipients	Solubility (mg/mL)
Oleic acid	0.92 ± 0.04
Capryol 90	4.45 ± 0.31
Maisine oil	0.32 ± 0.30
Labrafil M	6.58 ± 0.19
Lauroglycol FCC	4.59 ± 0.05
Tween 80	9.17 ± 0.07
Transcutol HP	14.1 ± 0.14
Polypropylene glycol	3.79 ± 0.62
Cremophor EL	9.05 ± 0.04
Peceol	2.17 ± 0.07

**Table 3:** Box-Behnken design (BBD) experimental conditions and responses on effect of independent variables on average droplet size, PDI, zeta potential, and drug content.

Run	Independent Variables			Responses			
	X_1_	X_2_	X_3_	Y_1_	Y_2_	Y_3_	Y_4_
	Capryol 90 (%*v*/*v*)	Tween 80 (%*v*/*v*)	Transcutol HP (%*v*/*v*)	Average Droplet Size (nm)	PDI	Zeta Potential (mV)	Drug Content (%)
1	7.5	5	10	323.6 ± 16.0	0.55 ± 0.03	−3.2 ± 0.2	96.3 ± 4.2
2	7.5	20	10	135.5 ± 1.77	0.28 ±0.05	−1.7 ± 0.3	90.12 ± 1.2
3	22.5	5	10	914.9 ± 84.3	0.45 ±0.35	−2.09 ± 0.3	95.85 ± 1.8
4	22.5	20	10	191.3 ± 3.60	0.28 ± 0.016	−1.67 ± 0.2	88.02 ± 3.9
5	15	5	5	807.4 ± 133	0.91 ± 0.08	−1.79 ± 0.1	98.43 ± 1.6
6	15	5	20	832.2 ± 161.4	0.94 ± 0.07	−1.86 ± 0.1	82.23 ± 2.3
7	15	20	5	143.6 ± 4.34	0.51 ±0.01	−1.85 ± 0.22	70.9 ± 0.5
8	15	20	20	124.1 ± 1.34	0.32 ± 0.045	−1.52 ± 0.3	101.4 ± 2.9
9	7.5	10	5	167.2 ± 5.25	0.36 ± 0.023	−1.85 ± 0.2	93.4 ± 1.01
10	22.5	10	5	1114.0 ± 69.5	0.96 ± 0.05	−1.83 ± 0.9	91.1 ± 0.74
11	7.5	10	20	118.8 ± 1.38	0.32 ± 0.004	−1.93 ± 0.4	104.0 ± 0.9
12	22.5	10	20	176.5 ± 3.8	0.26 ± 0.20	−2.67 ± 0.9	93.3 ± 4.5
13	15	10	10	311.9 ± 40.9	0.47 ± 0.08	−2.63 ± 1.4	98.19 ± 1.3
14	15	10	10	233.9 ± 15.6	0.30 ± 0.015	−3.14 ± 0.8	92.7 ± 2.1
15	15	10	10	330.6 ± 25.5	0.55 ± 0.01	−4.2 ± 1.3	89.0 ± 1.8

**Table 4:** Summary of results of regression analysis for responses Y_1_, Y_2_, Y_3_ and Y_4_. * *p* < 0.05.

Variables	Y_1_	Y_2_	Y_3_	Y_4_
	Average Droplet Size (nm)	PDI	Zeta Potential (mV)	Drug Content (%)
	Analysis of Variance *p*-Values			
X_1_ = % Oil (Capryol 90)	0.025 *	0.688	0.618	0.204
X_2_ = % Surfactant (Tween 80)	0.007 *	0.061	0.047 *	0.086
X_3_ = % Co-surfactant (Transcutol HP)	0.119	0.136	0.974	0.049 *

**Table 5:** Effective permeability of A190-PD-60, A190-PD-60 dispersion and A190-PD-60-ME. Data is represented as Mean ± SD (n = 5–6). BLD = below limit of detection. *** *p* < 0.001, compared to A190-PD-60 in dispersion.

Sample	Effective Permeability Across Artificial Membrane (P_e_, ×10^−6^, cm/s)
A190-PD-60 in PBS	BLD
A190-PD-60 dispersion	0.1 ± 0.04
A190-PD-60-ME	10.8 ± 1.76 ***

**Table 6:** Plasma pharmacokinetic parameters of A190-PD-60 following oral administration of A190-PD-60 dispersion and A190-PD-60-ME to rats. Data is represented as Mean ± SEM; n = 4–5.

Formulation	A190-PD-60 Dispersion	A190-PD-60-ME
Dose (mg/kg)	20	20
t_max_ (h)	31.5 ±1.5	1.7 ± 0.3
t_1/2_ (h)	14.11 ± 1.94	9.32 ± 1.27
C_max_ (ng/mL)	26.47 ± 0.55	438.85 ± 74.55
AUC_0→t_ (ng/mL × h)	828.10 ± 102.86	4914.33 ± 525.79
AUC_0→inf_ (ng/mL × h)	930.98 ±130.30	4974.52 ±521.22
Relative Bioavailability (%)	100	593

## Data Availability

All data needed to evaluate the conclusion are presented in the paper and/or the Supplementary Materials. The data that support the findings of this study are available upon reasonable request.

## Supplementary Materials

The following supporting information can be downloaded at: https://www.mdpi.com/article/10.3390/biom15081101/s1, Figure S1: ^1^H and ^13^C NMR Spectrum of A190-PD-9, Figure S2: ^1^H and ^13^C NMR Spectrum of A190-PD-14, Figure S3: ^1^H and ^13^C NMR Spectrum of A190-PD-154, Figure S4: ^1^H and ^13^C NMR Spectrum of A190-PD-60, Figure S5: Ex-vivo hydrolysis of prodrugs in rat plasma, Figure S6: Representative images of A190-PD-60-ME formulations as suggested by Box-Behnken analysis, Figure S7: In vitro cell cytotoxicity assay of A190-PD-60 (in 0.2% DMSO), vehicle microemulsion, and A190-PD-60-ME on HepG2 cells and Caco-2 cells after incubation for 48 h. Table S1: Aqueous solubility of A190 prodrugs.
