# Supplementary material for: Oral Pharmacokinetic Evaluation of a Microemulsion-Based Delivery System for Novel A190 Prodrugs
Source: Biomolecules. 2025 Jul 30;15(8):1101. doi: 10.3390/biom15081101 (PMC12383972; doi:10.3390/biom15081101)
Supplement: Supplementary file 1 [file biomolecules-15-01101-s001.zip › biomolecules-3769944-supplementary.pdf]

Supplementary materials

Oral Pharmacokinetic Evaluation of a Microemulsion-Based Delivery System for Novel A190 Prodrugs

Sagun Poudel[1], Chaolong Qin[1], Rudra Pangeni[1], Ziwei Hu[2], Grant Berkbigler[2], Madeline Gunawardena[1], Adam S. Duerfeldt[2,*], Qingguo Xu[1,3,*]

[1]Department of Pharmaceutics, Virginia Commonwealth University, Richmond, VA, USA

[2]Department of Medicinal Chemistry, University of Minnesota, Minneapolis, MN, USA

[3]Department of Ophthalmology, Department of Pediatrics, Department of Biomedical Engineering, Massey Cancer Center, Center for Pharmaceutical Engineering, and Center for Drug Discovery, Virginia Commonwealth University, Richmond, VA, USA

Corresponding authors: Qingguo Xu (qxu@vcu.edu) and Adam S. Duerfeldt (aduerfel@umn.edu)

Table S1: Aqueous solubility of A190 prodrugs

Compound	Aqueous solubility (µg/mL)	Chemical Name *
A190	70±1.34	3-((4-((4-fluorobenzyl)oxy)-3-methylbenzyl)amino)benzoic acid
A190-PD-9	3±0.43	methyl 3-((4-((4-fluorobenzyl)oxy)-3-methylbenzyl)amino)benzoate
A190-PD-14	17±2.12	ethyl 3-((4-((4-fluorobenzyl)oxy)-3-methylbenzyl)amino)benzoate
A190-PD-60	1.4±0.23	(5-methyl-2-oxo-1,3-dioxol-4-yl)methyl 3-((4-((4-fluorobenzyl)oxy)-3-methylbenzyl)amino)benzoate
A190-PD-154	15.5±1.03	isopropyl 3-((4-((4-fluorobenzyl)oxy)-3-methylbenzyl)amino)benzoate

* Chemical names of A190 and prodrugs of A190 were derived from ChemDraw.

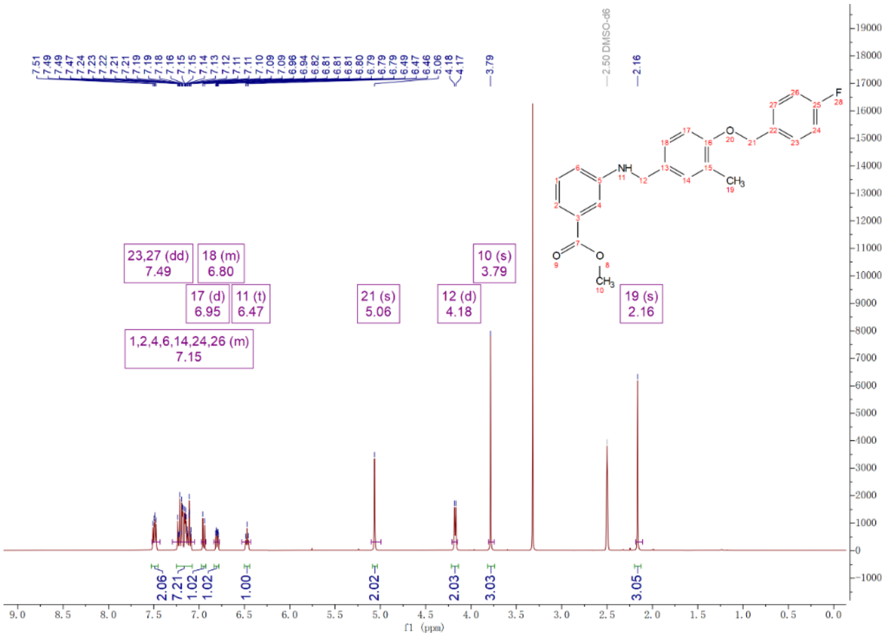

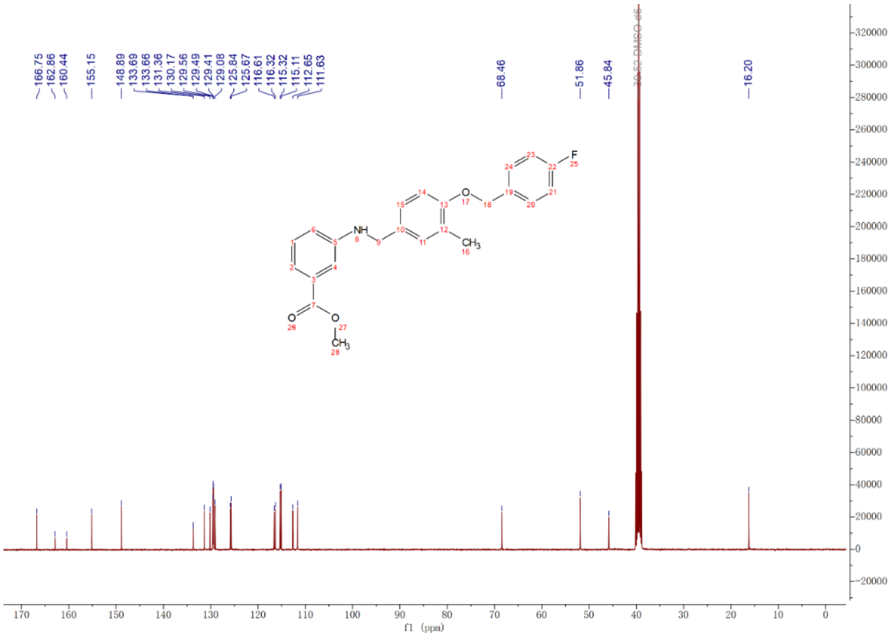

Fig S1: ^{1}H and ^{13}C NMR Spectrum of A190-PD-9

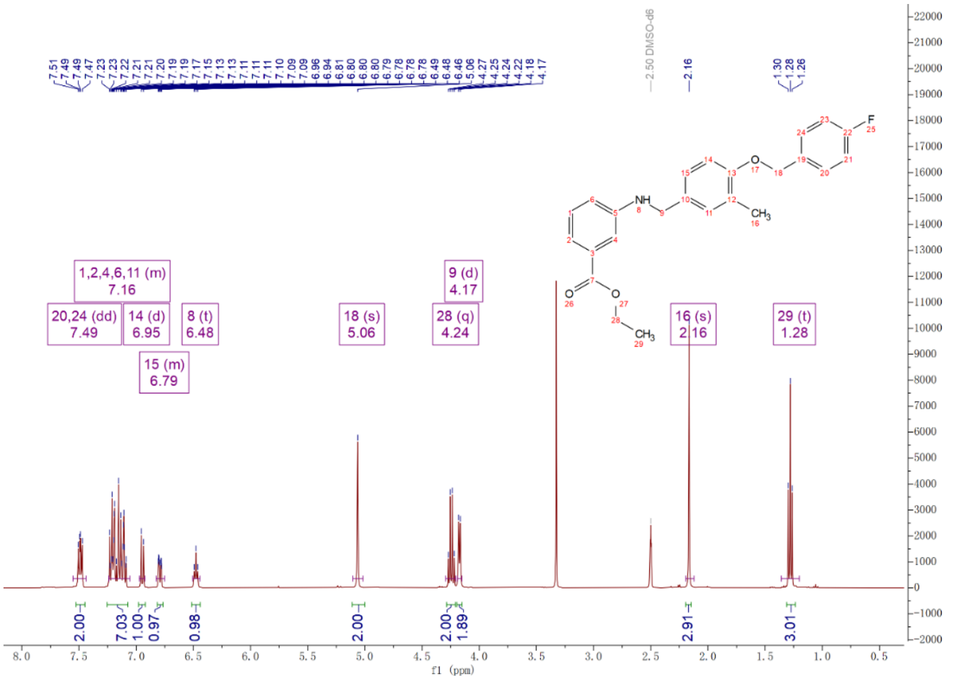

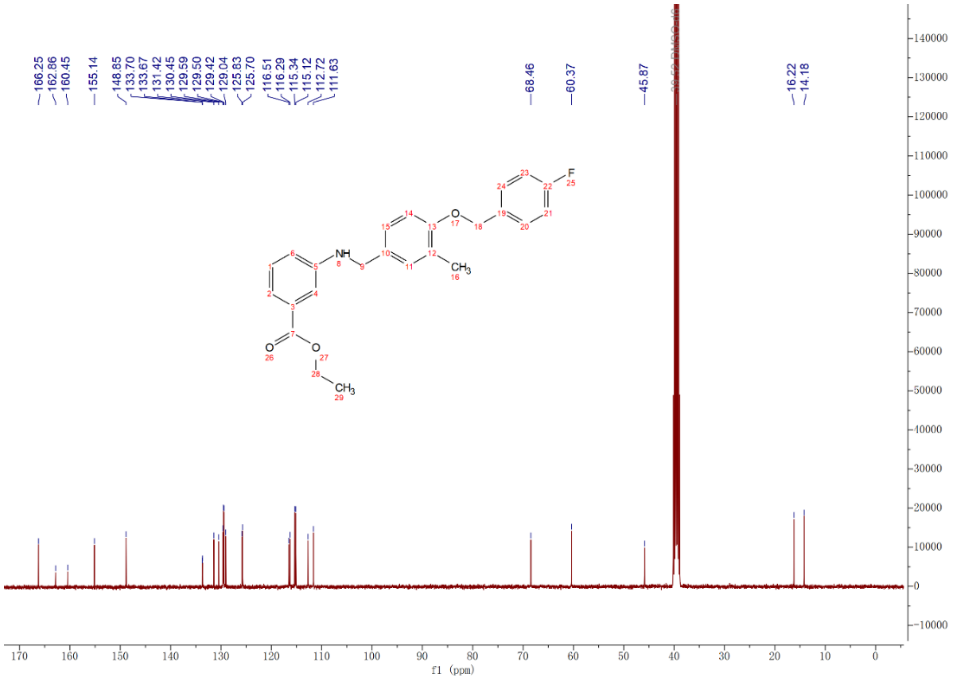

Fig S2: ^{1}H and ^{13}C NMR Spectrum of A190-PD-14

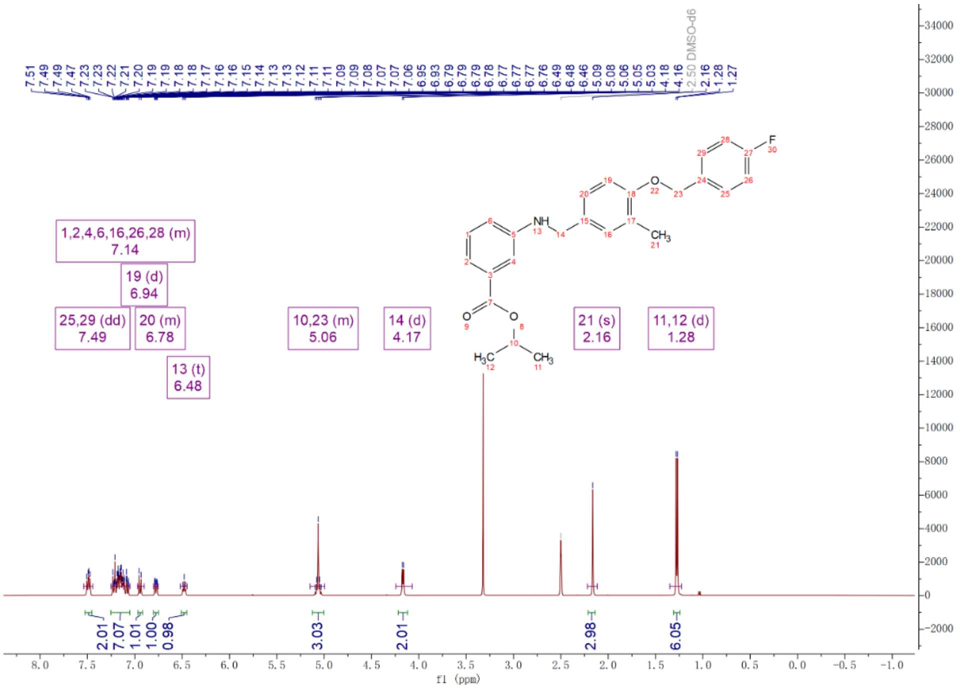

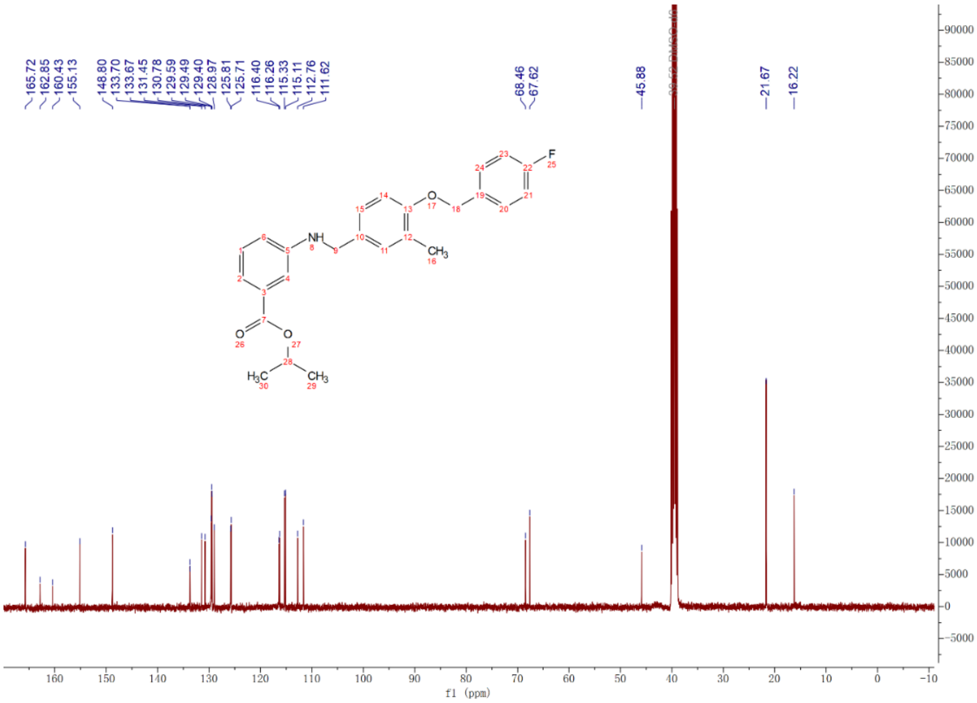

Fig S3: ^{1}H and ^{13}C NMR Spectrum of A190-PD-154

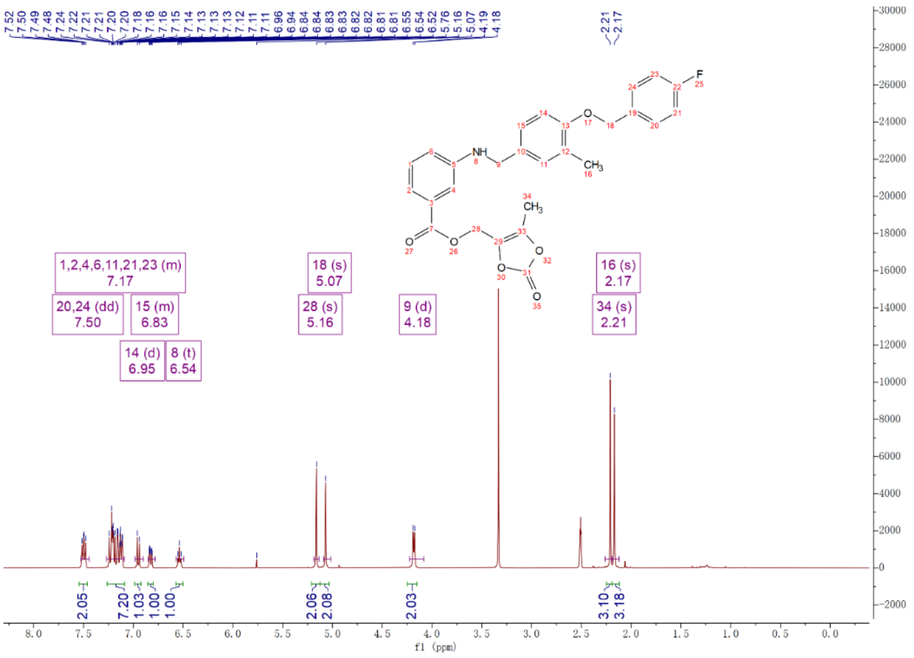

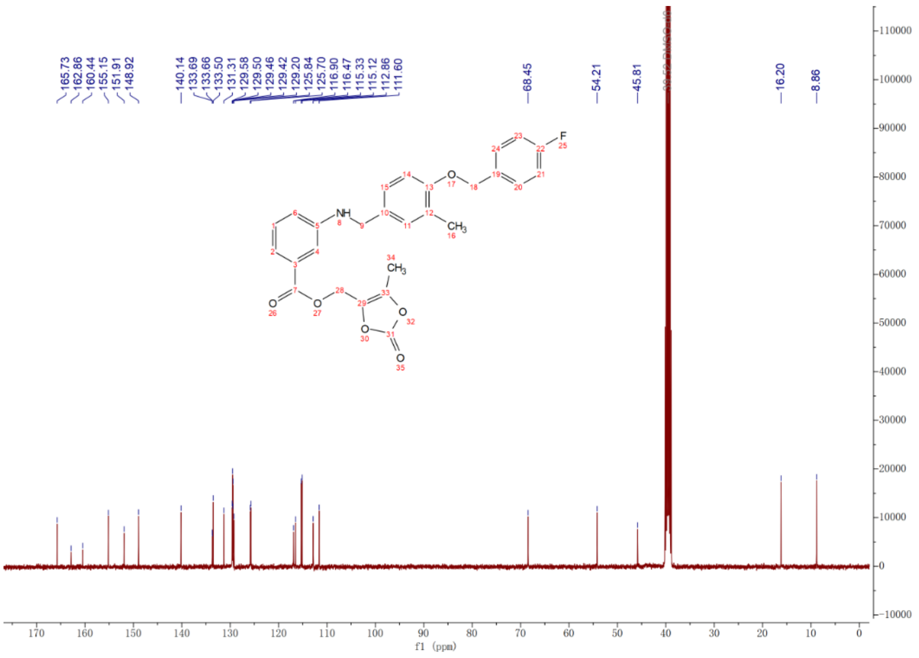

Fig S4: ^{1}H and ^{13}C NMR Spectrum of A190-PD-60

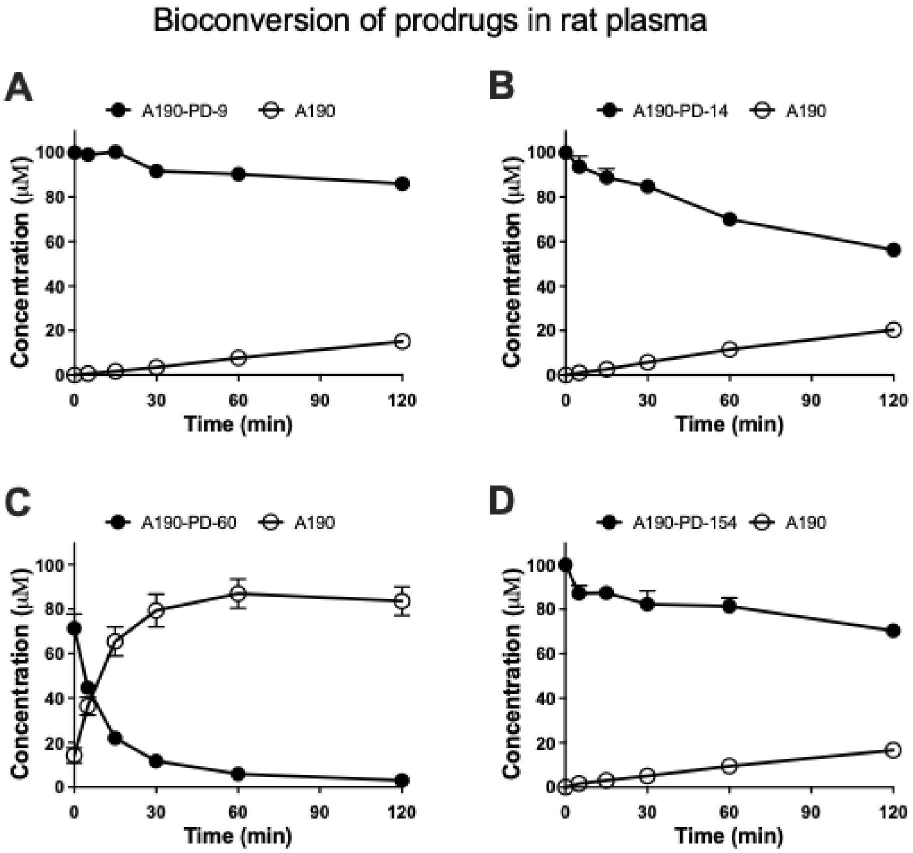

Figure S5: Ex-vivo hydrolysis of prodrugs in rat plasma. Concentration-time profiles of degradation of (A) A190-PD-9, (B) A190-PD-14, (C) A190-PD-60, and (D) A190-PD-154 and conversion into parent drug A190 in rat plasma. Each prodrug (100 μM) was incubated with rat plasma at 37°C for 120 min.

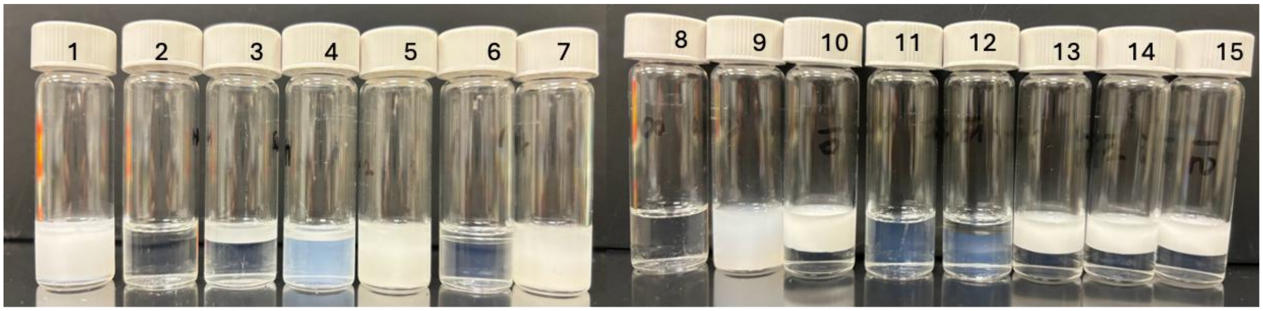

Figure S6: Representative images of A190-PD-60-ME formulations as suggested by Box-Behnken analysis. Formulation #8 was used for permeability, cell viability, stability and oral pharmacokinetic studies.

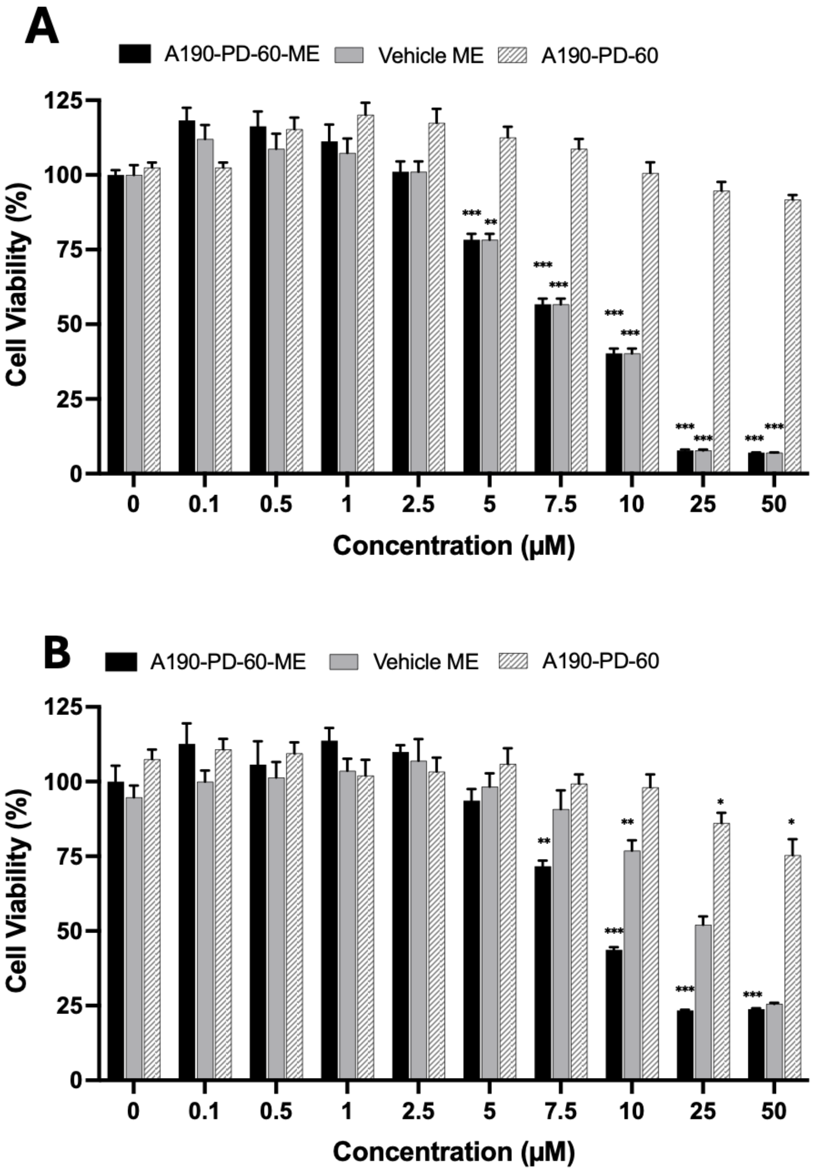

Figure S7: *In vitro* cell cytotoxicity assay of A190-PD-60 (in 0.2% DMSO), vehicle microemulsion, and A190-PD-60-ME on (A) HepG2 cells and (B) Caco-2 cells after incubation for 48 h. Values are mean ± SD (n=5). *$p < 0.05$, **$p < 0.01$, ***$p < 0.001$ compared to respective DMEM or 0.2% DMSO controls.
